# Experimental Model of Human Malignant Mesothelioma in Athymic Mice

**DOI:** 10.3390/ijms19071881

**Published:** 2018-06-26

**Authors:** Didier J. Colin, David Cottet-Dumoulin, Anna Faivre, Stéphane Germain, Frédéric Triponez, Véronique Serre-Beinier

**Affiliations:** 1 MicroPET/SPECT/CT Imaging Laboratory, Centre for BioMedical Imaging (CIBM), University Hospitals and University of Geneva, 1211 Geneva 4, Switzerland; didier.colin@unige.ch (D.J.C.); stephane.germain@hcuge.ch (S.G.); 2 Department of Thoracic and Endocrine Surgery, University Hospitals and University of Geneva, 1211 Geneva 4, Switzerland; David.Cottet-Dumoulin@unige.ch (D.C.-D.); anna.faivre@unige.ch (A.F.); frederic.triponez@hcuge.ch (F.T.)

**Keywords:** cancer, pleura, mesothelioma, orthotopic xenotransplantation, athymic mouse, immune cells

## Abstract

Malignant pleural mesothelioma (MPM) is a thoracic aggressive cancer caused by asbestos exposure, which is difficult to diagnose and treat. Here, we characterized an in vivo orthotopic xenograft model consisting of human mesothelioma cells (designed as H2052/484) derived from a pleural NCI-H2052 tumor injected in partially immunodeficient athymic mice. We assessed tumor formation and tumor-dependent patterns of inflammation. H2052/484 cells conserved their mesothelioma phenotype and most characteristics from the parental NCI-H2052 cells. After intra-thoracic injection of H2052/484 cells, thoracic tumors developed in nearly all mice (86%) within 14 days, faster than from parental NCI-H2052 cells. When the mice were euthanized, the pleural lavage fluid was examined for immune cell profiles. The pleural immune cell population increased with tumor development. Interestingly, the proportion of myeloid-derived suppressor cell and macrophage (especially CD206^+^ M2 macrophages) populations increased in the pleural fluid of mice with large mesothelioma development, as previously observed in immunocompetent mice. This reliable orthotopic model recapitulates human mesothelioma and may be used for the study of new treatment strategies.

## 1. Introduction

Malignant pleural mesothelioma (MPM) is an aggressive tumor that develops in the lining of the lungs. This cancer is causally associated with asbestos exposure. Although asbestos use is banned in many of the world’s industrialized countries, the incidence of mesothelioma has overall not decreased for the last twenty years in most occidental countries [1]. Surgery is an option for early-stage MPM patients but not for most patients with advanced invasive disease [2] for whom treatment consists of palliative chemotherapy combining cisplatin with pemetrexed. While this treatment may relieve symptoms, it provides only modest survival, since the median survival average is 9–18 months from the time of diagnosis. Therefore, there is an urgent need for more effective treatments. Previous results from our laboratory, mostly obtained from in vitro experiments, suggest that inhibition of the Macrophage migration inhibitory factor (MIF)/CD74 pathway decreases the development of MPM [3]. These data need validation in a reliable in vivo preclinical model. Several murine mesothelioma models have been developed, and the selection of an appropriate model depends upon the experimental aims. Asbestos-induced and genetically engineered mesothelioma mouse models recapitulate the phenotypic and genetic heterogeneity as well as the carcinogenesis steps of human mesothelioma. They have also a strong predictive power for drug response and resistance, but their use to validate new therapies is limited by a low take rate, a long latency in tumor development, and a high cost. Syngeneic transplantation of murine mesothelioma cell lines could be an alternative with a high take rate and a rapid tumor development. Nevertheless, murine and human cells present fundamental phenotypic and functional differences. For example, there are two CD74 isoforms in mice (p31 and p41) and four in humans (p33, p35, p41, and p43) [4]. Up to now, the role of these different isoforms has not been clearly identified. Therefore, the effect of a treatment on murine mesothelial tumors could be not reproducible in human mesothelioma. Finally, preclinical studies on MPM mostly rely on subcutaneous or peritoneal xenotransplants of human mesothelioma cell lines in immunodeficient mice. These models provide reliable data and allow for rapid clinical translation. The major limitation is that the tumor environment is different from the in situ thoracic pleural mesothelioma environment. Transplantation in the orthotopic site offers a tumor microenvironment close to that of the original human tumor. To date, the use of the orthotopic thoracic site for xenografting has not been widespread, which is largely due to the technical difficulties in reaching and monitoring tumor development in this location. Here, we present a reliable orthotopic model of human MPM obtained after injection of a human mesothelioma cell line into the pleural space of athymic mice. Athymic mice have the advantage to be only partially immunodeficient, since they lack the thymus but produce most other immune cell types. Phenotypical and molecular characterizations of the tumor masses are described. For the first time in this orthotopic xenograft mesothelioma model, immune cell populations in the pleural environment of human mesothelioma-bearing mice are assessed.

## 2. Results

### 2.1. Selection and Characterization of the Human H2052/484 Cell Line

We were interested in the human MPM H2052 cells. Previous data from our lab showed that H2052 cells expressed MIF and its receptor CD74 [3] and that reduction of MIF and CD74 expression decreased the growth of H2052 cells. We previously observed that H2052 cells injected into the pleural cavity of athymic nude mice formed extensive pleural tumors in nearly all injected mice (5/6). Nevertheless, H2052 tumors developed slowly in the thoracic cavity. Using 2-deoxy-2-[^18^F]fluoro-D-glucose ([^18^F]FDG)-PET/CT analyses, H2052 pleural tumors were identified in the thoracic cavity starting on day 69 after cell injection, and their development continued until 102 days after cell injection, time at which the mice were sacrificed because of the extent of tumor development [3]. In order to evaluate whether preliminary in vivo engraftment of H2052 cells increased their tumorigenicity, thoracic tumors were mechanically dissociated into cell suspensions. Among many cell populations which could be maintained in monolayer culture, one cell line named H2052/484 was further characterized.

First, we compared the vitality and the multiplication of H2052/484 cells to those of the parental H2052 cells and of three other MPM cell lines using a mitochondrial activity assay (3-(4,5-dimethylthiazol-2-yl)-2,5-diphenyltetrazolium bromide, MTT) and crystal violet assay, respectively.

**Figure 1:**
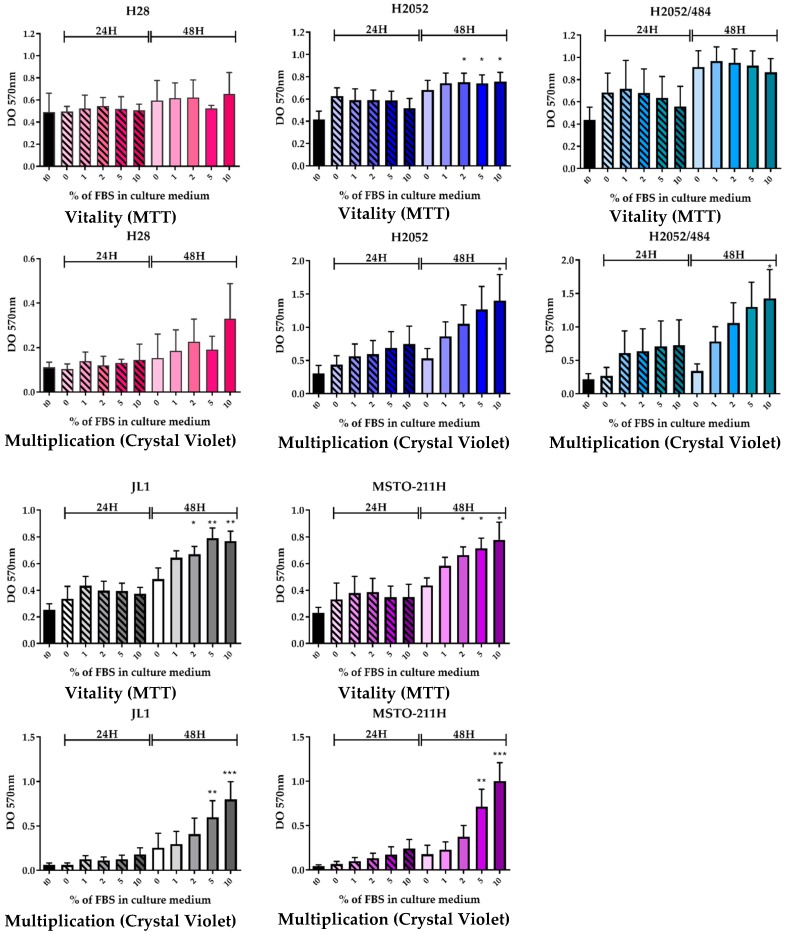
Cell vitality (3-(4,5-dimethylthiazol-2-yl)-2,5-diphenyltetrazolium bromide, MTT) and multiplication (Crystal Violet) of H2052/484 cells (blue-green) are similar to those of the parental H2052 cells (blue). The vitality and multiplication of the five malignant pleural mesothelioma (MPM) cell lines (H28 in pink; H2052 in blue; H2052/484 in blue-green; JL-1 in grey, and MSTO-211H in purple) were evaluated after the cells were cultured for 24 h (hashed bar) and 48 h (full bar) in medium supplemented with different percentages of fetal bovine serum (FBS). DO, optical density. The bars are mean values (±SEM) for *n* = 3–7 experiments. Kruskal–Wallis test between FBS concentrations and 0%: * *p* < 0.05, ** *p* < 0.01, *** *p* < 0.001.

We observed similar vitality and multiplication rates for H2052/484 and the parental H2052 cells (Figure 1). Nutritional supplementation with fetal bovine serum (FBS) did not change the vitality of H28, H2052/484, and H2052 cells after 48 h of culture (Figure 1, upper panels); however, the multiplication of these three cell lines increased dose-dependently with FBS concentrations after 48 h of culture. After 48 h, the density of cells cultured with 10% FBS compared to cells cultured with 0% FBS, estimated by the absorbance level, was 2.81 ± 0.94 times higher for H28 (*n* = 3), 2.99 ± 0.80 times higher for H2052 (*n* = 7), and 6.53 ± 3.10 times higher for H2052/484 (*n* = 7). FBS supplementation dose-dependently increased the vitality of JL-1 and MSTO-211H as well as their multiplication (Figure 1, left lower panels). After 48 h, the vitality of cells cultured with 10% FBS compared to cells cultured with 0% FBS was 1.65 ± 0.23 times higher for JL1 (*n* = 4) and 1.79 ± 0.25 times higher for MSTO-211H (*n* = 3). The density of cells cultured for 48 h with 10% FBS compared to cells cultured with 0% FBS, estimated by the absorbance level, was 7.60 ± 0.07 times higher for JL1 (*n* = 3) and 12.23 ± 0.60 times higher for MSTO-211H (*n* = 3).

**Figure 2:**
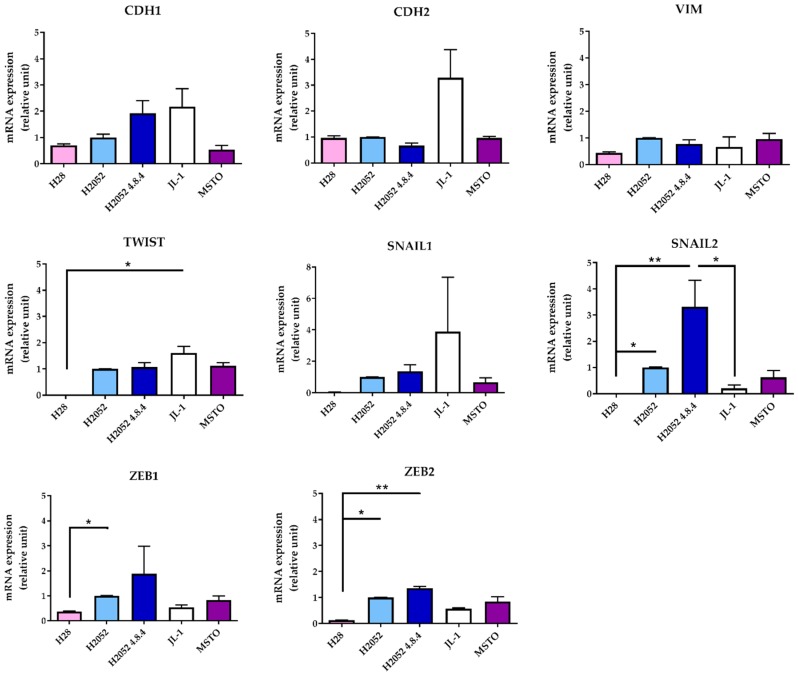
H2052/484 MPM cells express high levels of epithelial–to-mesenchymal (EMT) transcription factors. The mRNA levels of the EMT markers were measured in parental H2052 cells, in H2052/484 cells, and in three other MPM cell lines (H28, JL-1, and MSTO). The relative mRNA expression levels were measured by RT-qPCR and are presented as a ratio to the mRNA levels in parental H2052 cells. The data represent the mean values (±SD) of three independent experiments. Kruskal–Wallis test between MPM cell lines: * *p* < 0.05, ** *p* < 0.01.

Then, we compared the phenotype of H2052/484 cells to that of the parental H2052 cells and of three other MPM cell lines by studying the expression of different epithelial-to-mesenchymal (EMT) markers. Compared to parental H2052 cells, H2052/484 cells expressed 1.9 higher mRNA levels of the epithelial marker E-cadherin (CDH1) (Figure 2) and higher mRNA levels of the transcription factors SNAIL2 (3.3-fold change), ZEB1 (1.9-fold change), and ZEB2 (1.4-fold change), which are considered mesenchymal markers.

**Figure 3:**
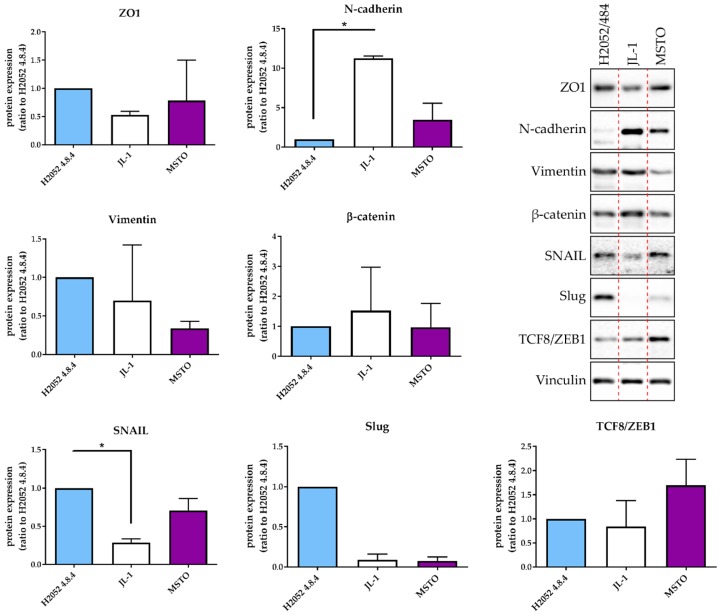
H2052/484 cells express epithelial and mesenchymal markers. Protein expression of EMT markers was measured in H2052/484 cells and two other MPM cell lines (JL-1 and MSTO) by western blotting. Representative western blot results are shown; the dashed red lines indicate the manual cropping of the bands detected for samples run on the same gels and identically exposed. Protein expression levels are presented as the ratio to the respective protein level in H2052/484 cells. The data represent the mean values (±SD) of three independent experiments. Kruskal–Wallis test between MPM cell lines: * *p* < 0.05.

The mRNA expression levels of these three transcription factors were higher in H2052/484 cells compared to the three other MPM cell lines (H28, JL-1, and MSTO). These differences were not statistically significant. Interestingly, H28 cells failed to form tumors in vivo [3] and expressed the lowest mRNA levels of ZEB1, ZEB2, SNAIL1, SNAIL2, and TWIST. H2052/484 cells expressed the lowest level of N-cadherin mRNA (CDH2). Western blot analyses of EMT markers in H2052/484, JL-1, and MSTO cell lines confirmed the highest expression levels of Snail (SNAIL1) and Slug (SNAIL2) and the lowest expression of N-cadherin in H2052/484 cells (Figure 3). We did not detect E-cadherin protein expression in any of the studied MPM cell lines. MIF and CD74 mRNA levels in H2052/484 cells were similar to the levels in parental H2052 (for MIF: 1.39 ± 0.07, *n* = 3, for H2052/484; 1.31 ± 0.05, *n* = 3, for H2052; for CD74: 1.14 ± 0.07, *n* = 3, for H2052/484; 1.22 ± 0.22, *n* = 3, for H2052).

### 2.2. Characterization of Orthotopic Tumor Masses Generated by Human MPM H2052/484 Cells

**Figure 4:**
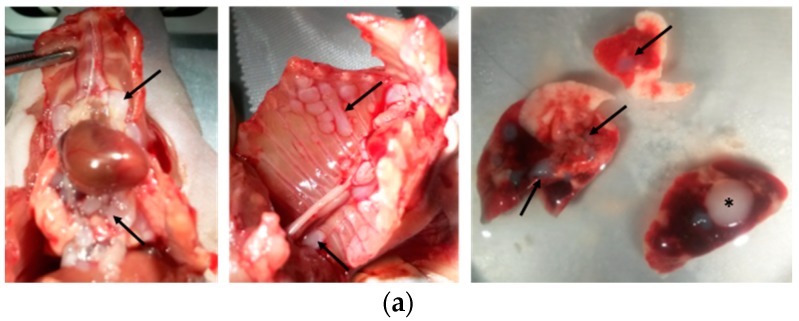

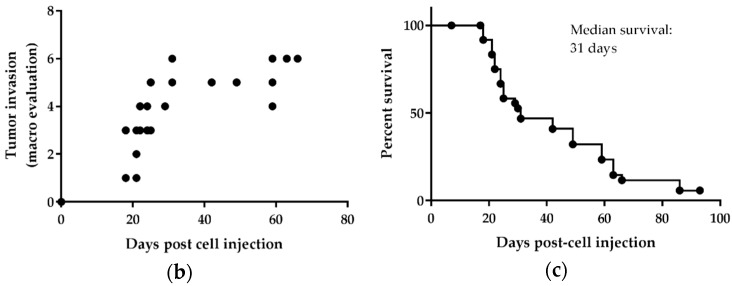
H2052/484 cells formed pleural mesothelioma in athymic mice. H2052/484 cells were injected intrapleurally (i.pl.) (1 × 10^6^ cells) into athymic nude mice (single experiment; *n* = 28). (**a**) H2052/484 tumors were free in the thoracic cavity or attached to the aortic arch (close to the thymus rudiment, left panel, black arrows), the inferior vena cava (left panel), thoracic muscles (middle panel), or to lungs (right panel). (**b**) The mice were sacrificed at different time points (end-point criteria), and the tumor scores were evaluated post-mortem following criteria described in Table 1. (**c**) Survival of athymic mice after intrapleural injection of H2052/484 MPM cells.

Intrapleural (i.pl.) injection of H2052/484 cells into athymic nude mice yielded sizable tumor masses identifiable by ([^18^F]FDG)-PET/CT imaging within 2 weeks. H2052/484 tumors developed in nearly all injected mice (24/28). The tumors were distributed freely in the thoracic cavity or attached to the aortic arch (close to the thymus rudiment), to the inferior vena cava, to thoracic muscles, or to the lungs (Figure 4a).

The tumor attached to the left lung (Figure 4, right panel, asterisk) was localized close to the injection site. These tumors were poorly invasive and did not often penetrate deep into intercostal tissues or into the lung to which they were attached. There was no evidence of metastases, as we found no tumors in distant organs. The mice were followed using positron-emission tomography/computerised tomography (PET/CT) imaging until they were sacrificed, once tumor development reached euthanasia endpoints (described in Material and Methods) such as large size or signs of unacceptable pain and clinical distress. Macroscopic evaluation of tumor size and extent was performed when the mice were sacrificed (Figure 4b). The scoring of tumor development is detailed in Table 1. Between days 18 to 29, tumors of different size and extent were observed, ranging from score 1 to 5 and indicating an active phase of tumor growth (Figure 4b). Between days 29 to 66, all tumors reached the maximum scores of 5 or 6 (Table 1). Half of the mice were sacrificed during the active phase of tumor growth, and a median survival of 31 days was observed (Figure 4c).

**Figure 5:**
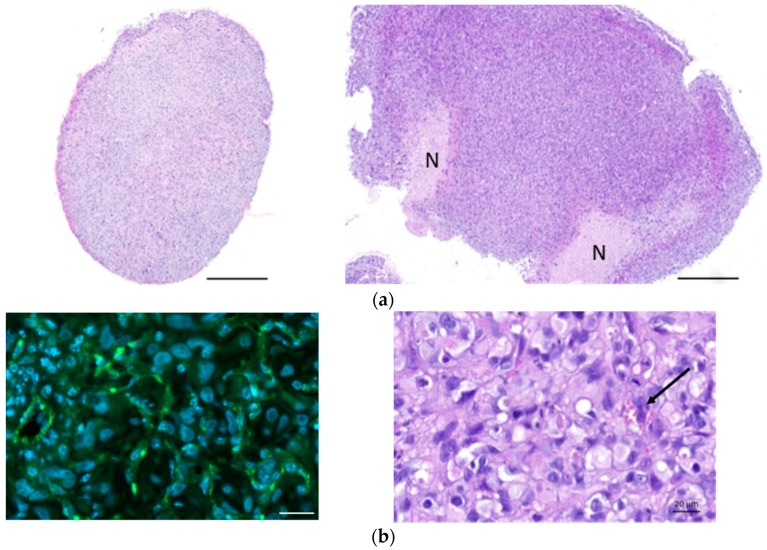

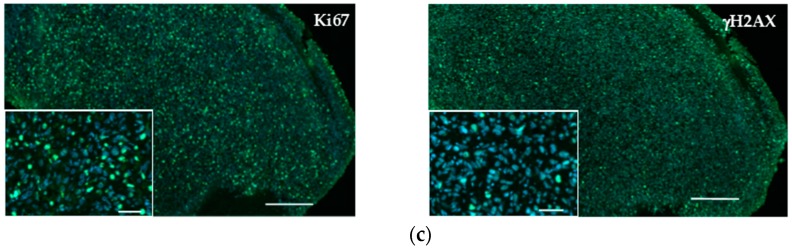
Characterization of H2052/484-derived orthotopic tumors. (**a**) Haematoxylin–eosin (HE) sections of two representative pleural MPM generated after intrathoracic injection of H2052/484 cells in athymic nude mice at day 46 after cell injection. Necrotic areas (N) were identified in the largest tumor (right panel). Scale bars: 500 μm. (**b**) Vascularization of H2052/484 tumors: left, CD31 (green, endothelial marker) expressed in tumors; right, HE staining showing red blood cells in vessels (black arrow) in the tumor. Scale bars: 20 μm. (**c**) Cell proliferation and DNA damage representative of cell apoptosis were identified in H2052/484 tumors. The tumor slices were stained (green) with anti-Ki67 (cell proliferation), anti-γ-H2AX (DNA damage and cell apoptosis), and DAPI (nuclear counterstaining, blue). Scale bars: 500 μm and 50 μm.

**Figure 6:**
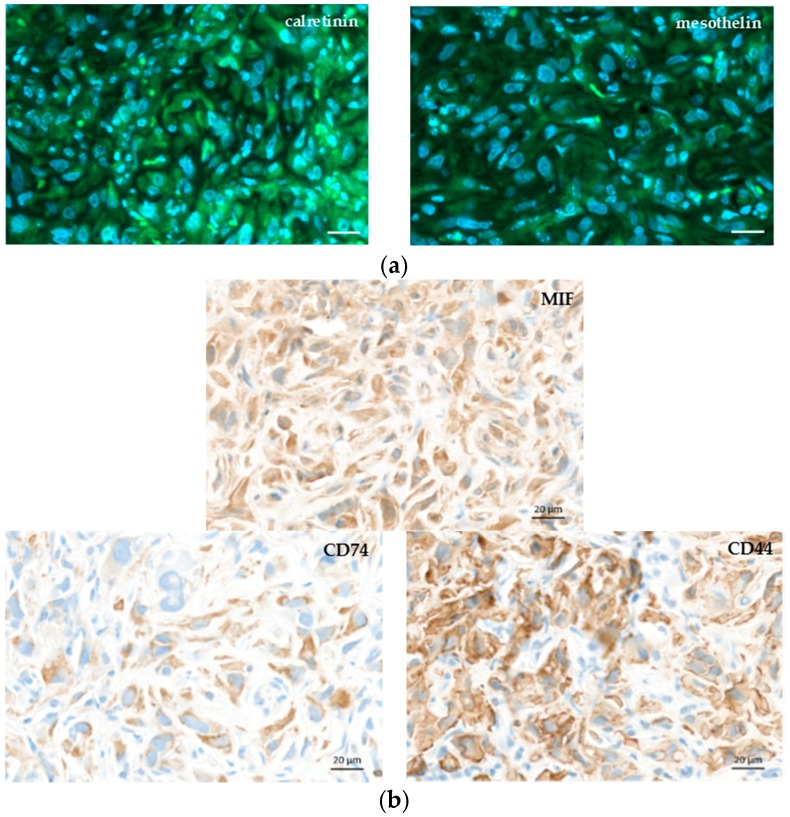
MPM markers and macrophage migration inhibitory factor (MIF), CD74, and CD44 expressions in orthotopic H2052/484 tumors. Representative photomicrographs of a H2052/484 intrathoracic tumor stained with (**a**) anti-calretinin, and anti-mesothelin, (**b**) anti-MIF, anti-CD74, and anti-CD44 antibodies. Scale bars: 20 μm.

Figure 5a shows haematoxylin–eosin (HE) staining of explanted and formalin-fixed H2052/484 pleural tumors. Necrotic (N) areas were observed in big tumors (mouse 2, right panel, Figure 5a). A meshwork of capillaries and vessels was detected inside the tumors by identifying the red blood cells on HE-stained slices (Figure 5b) or after immunostaining with anti-CD31 antibody (Figure 5b). Ki67 labelling showed a high proliferation rate of tumoral cells (Figure 5c). Apoptotic cells were also clearly identified after immunolabelling of γ-H2AX histone that showed strong homogeneous nuclear labelling (Figures 5c). The excised H2052/484 tumors were tested for retention of classical MPM markers. H2052/484 tumoral cells expressed calretinin and mesothelin (Figure 6a), confirming their MPM behaviour after in vivo orthotopic engraftment. Finally, cytoplasmic MIF was clearly observed in all H2052/484 tumor cells (Figure 6b). MIF receptor CD74 and co-receptor CD44 were also detected in the cytoplasm and the membrane of MPM cells (Figure 6b), suggesting an active MIF/CD74 pathway.

### 2.3. Local Immune Cell Response of Athymic Nude Mice Developing Orthotopic H2052/484 Tumors

**Figure 7:**
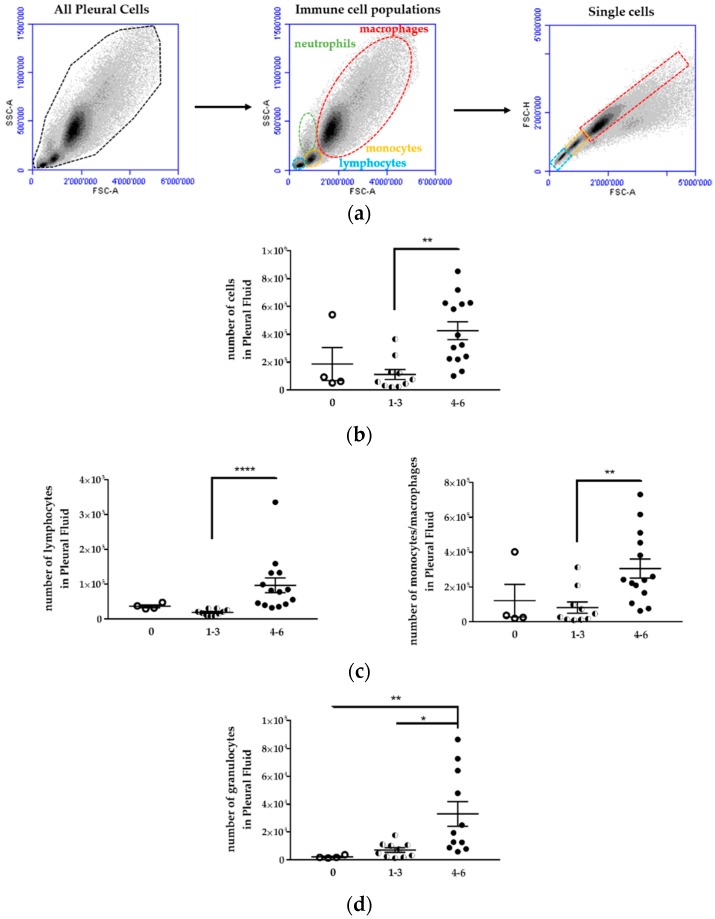
Immune cell populations increased in the pleural fluid of H2052/484 tumor-bearing mice. (**a**) Viable immune cell populations (lymphocytes (blue), monocytes (yellow), macrophages (red) and granulocytes (green)) were identified first according to side scatter (SSC-A) vs. forward scatter (FSC-A). Doublets were excluded using a pulse geometry gate FSC-H × FSC-A plot. Representative flow cytometry dot plots and scatter plots of pleural cells are shown. Comparisons of the number of (**b**) total cells, (**c**) lymphocytes, monocytes/macrophages, and (**d**) granulocytes in the pleural fluid of mice with no tumor (empty dots) and in tumor-bearing mice scored 1 to 3 (1–3; half dots) and 4 to 6 (4–6, full dots). The data represent the mean values ± SEM. Comparisons were made using Kruskal–Wallis test; * *p* < 0.05, ** *p* < 0.01, **** *p* < 0.0001.

Due to the lack of thymus maturation, a T-cell deficiency was observed in athymic nude mice. B cells, dendritic cells, and granulocytes were all relatively intact, and there was a compensatory increase in both natural killer (NK)-cell activity and tumoricidal macrophages in these mice (as reviewed in reference [5]). Therefore, an immune response could be expected after malignant cell injection. Using flow cytometry, the immune cell populations (lymphocytes, neutrophils, monocytes, and macrophages) were characterized in the thoracic cavity of mice injected i.pl with H2052/484 cells (Figure 7).

First, the cell populations were identified according to their sizes (forward scatter, FSC) and internal structures (side scatter, SSC). Second, labelling of immune cells with specific antibodies was performed as follows: CD19^+^B220^+^ for B lymphocytes, CD19^−^CD11b^+^ for monocytes/macrophages, CD19^−^CD11b^+^ F4/80^+^ for macrophages, CD11b^+^F4/80^+^CD206^+^ for M2 macrophages, CD49b^+^ for NK cells, CD11b^+^Gr1^+^ for myeloid-derived suppressor cells (MDSC).

Mice with a high tumor development score (4 to 6) showed an increase of cell number in the pleura compared to mice with a low tumor development score (1 to 3) or mice without tumor (Figure 7b, Table 2). The number of cells for each cell population, i.e., lymphocytes, granulocytes (neutrophils), monocytes, and macrophages, increased in the pleural cavity of mice with a high tumor development score (Figure 7; Table 2).

**Figure 8:**
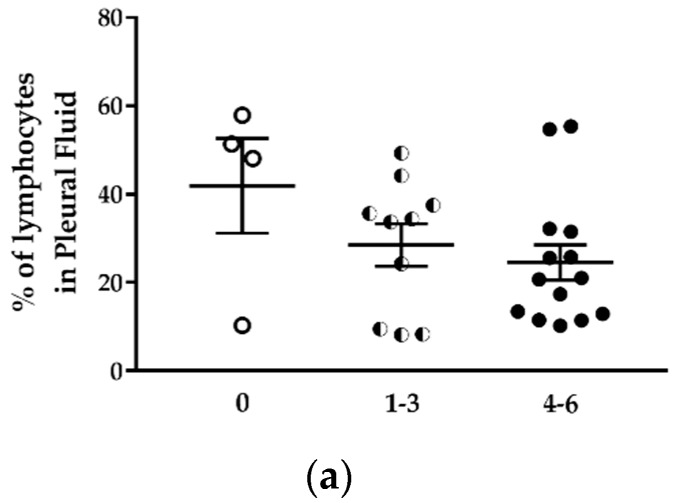

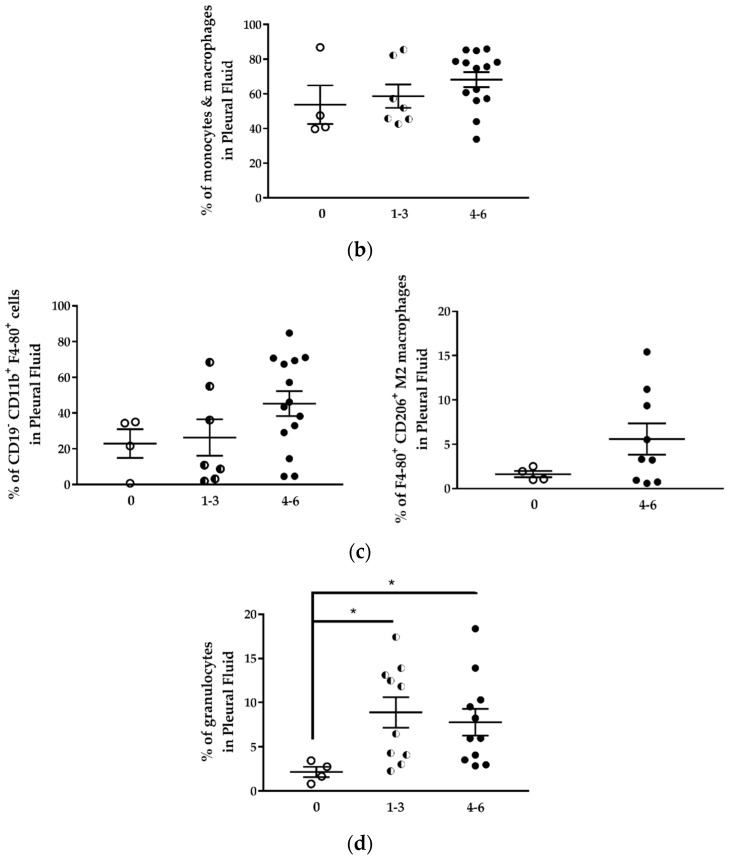
The proportion of immune cell populations in the pleural fluid of H2052/484 tumor-bearing mice changed with the tumor score. Comparisons of percentages of (**a**) total lymphocytes, (**b**) total monocytes/macrophages, (**c**) CD19^−^CD11b^+^F4/80^+^ macrophages and CD206^+^ M2 macrophages, and (**d**) granulocytes in the pleural fluid of mice with no tumor (empty dots) and in tumor-bearing mice with development scores 1–3 (half dots) and 4–6 (full dots). The percentages represent the number of each cell population in the total pleural cell number as determined by flow cytometry. The data represent the mean values ± SEM. Comparisons were made using Kruskal–Wallis test; * *p* < 0.05.

Despite the increased number of lymphocytes in the pleural cavity, the percentage of total lymphocytes in the pleural cell population tended to decrease with the increase of the tumor development score (Figure 8a; Table 3). The percentage of lymphocytes in mice with tumor score 4–6 was 1.7 lower than in mice without tumor. This lymphocyte population identified by the FSC and SSC cytometric parameters included T and B lymphocytes and NK cells. The percentage of CD19^+^B220^+^ B lymphocytes was relatively stable in mice with different tumor development scores (Table 3), representing 6.8 to 17.5% of total cells in the thoracic cavity. The monocytes/macrophages population represented 53.8 to 68.3% of pleural cells (Table 3). This percentage was not different in mice with or without tumor (Figure 8b). This population contains several cell types, including MDSC, dendritic cells, monocytes, macrophages. The percentage of CD11b^+^F4/80^+^ macrophages increased in the thoracic cavity of mice with high tumor development score (Figure 8c, left panel) from 22.9 ± 8.0% (mice without tumor) to 45.3 ± 7.0% (mice with tumor development score 4–6) (Table 3).

We observed an increase of the percentage of CD11b^+^F4/80^+^CD206^+^ M2 immunosuppressive macrophages in the pleural cell population of mice with higher tumor scores from 1.6% (mice without tumor) to 5.6% (mice with tumor development score 4–6) (Figure 8c, right panel; Table 3). This result is in concordance with previous reports of Jackaman C et al. [6].

Using an immunocompetent orthotopic mouse model of MPM, it was shown that the proportion of CD11b^+^F4/80^+^ tumor-associated macrophages increased significantly with MPM progression. Large tumors contained more macrophages of the M3 subset (a macrophage subset expressing a mixed M1 and M2 phenotype) and MDSCs. In this model, the percentage of CD11b^+^Gr1^+^ MDSC was two-fold higher in mice with tumors compared to mice without tumors (Table 3). Finally, the proportion of neutrophils in the pleural cell population was approximately fourfold higher in mice with tumors than in mice without tumors, and this independently of the tumor development score (Figure 8d; Table 3). While the proportion of total lymphocytes decreased with tumor development, we observed a sixfold increase in the neutrophil-to-lymphocyte ratio in mice with tumor development compared to mice without tumors (Table 3).

In summary, a high score (4–6) of H2052/484 tumor development in athymic mice was associated with an increase of immune cells in the thoracic cavity. Two-thirds of this population were CD11b^+^ cells, with a high proportion of F4/80^+^ macrophages and Gr1^+^ MDSC. Tumor development was also associated with a decrease of the percentage of total lymphocytes and an increase of the percentage of neutrophils.

## 3. Discussion

In order to assess the effect of the MIF/CD74 pathway in the development of MPM, we derived a new human MPM cell line expressing MIF, CD74, and CD44 and able to generate orthotopic intra-thoracic tumors. H2052/484 cells were obtained from the dissociation of a pleural tumor obtained after NCI-H2052 cell injection. Furthermore, H2052/484 cells conserved their mesothelioma phenotype and most characteristics of the parental H2052 cells. They demonstrated faster tumor growth than parental H2052 cells after intrathoracic injection in athymic mice. This higher tumor development may be related to higher levels of the EMT transcription factors Snail 2, Zeb1, and Zeb2. The activation of the EMT program is commonly observed in human cancers and is closely related to tumor invasiveness and progression [7]. H2052/484 cells were modestly virulent in vivo, and the mice were found to tolerate a certain level of tumor burden (1 × 10^6^ cells) over a two-week time course, without euthanasia requirements due to distress. Thus, this model represents a reproducible mean to test new therapies targeting the MIF/CD74 pathway as well as other pathways that promote the growth of MPM. This model provides a large time window to evaluate the anticancer effects of new treatments and possible tumor relapse and resistance due to subpopulations of cells that might escape therapy. We used athymic mice as hosts, given that our study objective was to evaluate the effects of the MIF/CD74 pathway inhibition on human MPM development in vivo. These mice are partially immunodeficient because of the lack of thymus [8], which leads to a very poor response to thymic-dependent antigens [5]. Except for the lack of T lymphocytes, most other immune cell types are present in these mice, and we observed an increase in the immune cell population in the pleural fluid with the increase of tumor development. CD19^+^B220^+^ B lymphocytes and NK cells (CD49b^+^) were identified in the pleural fluid. No expansion of these populations was observed in tumor-bearing mice compared to mice without tumors. Myeloid-derived suppressor cells (CD11^+^Gr1^+^), monocytes (CD19^−^CD11b^+^), and macrophages (CD19^−^CD11b^+^F4/80^+^) were also detected in the pleural fluid. Interestingly, MDSC and macrophages (especially CD206^+^ M2 macrophages) expanded during MPM development as previously shown in an immunocompetent mouse model of mesothelioma [6]. The lack of T cells in nude mice is not an obstacle to study the relationship between inflammatory cells and mesothelioma development. Indeed, Jackaman et al. have shown [6], using an immunocompetent mouse model, that the suppressive role of regulatory T cells is important during the early stages of mesothelioma tumor evolution, but, in advanced-stage mesothelioma, myeloid cells and macrophages are major regulatory cells, as confirmed in our study. Both cell types have been shown to promote tumor growth, recurrence, and tumor burden in multiple ways, including promotion of angiogenesis and immunosuppressive activity [9,10]. Several studies reported a role for macrophage migration inhibitory factor (MIF) in promoting MDSC and macrophage accumulation and immunosuppressive activity in several cancers [11,12]. We previously showed that human MPM expresses MIF and its receptor CD74 and that this pathway is important for MPM cells proliferation [3]. In this model, MIF secretion by H2052/484 tumor cells may attract immunosuppressive cells such as MDSC and polarized macrophages toward an immunosuppressive M2 phenotype, thus promoting tumor growth. We plan to evaluate the effect of MIF inhibitors on H2052/484 development. The data obtained on the extent of tumor development and the immune cell types present in the local (pleural) tumor environment should help us to design new therapies. Then, these new agents should be validated on other mouse models such as humanized mouse models. In these models, immunocompromised mice (generally non-obese diabetic (NOD) scid gamma and NOD Rag gamma mice characterized by a great immunodeficiency) are immunologically reconstituted with human immune cells. The effects of MIF inhibitors on the total human immune cell populations could be characterized.

In summary, this study shows that the orthotopic xenotransplantation model of H2052/484 MPM cells in nude mice is a reproducible model to study the functional and mechanistic effects of new treatments for MPM. This model can be used to test the therapeutic effects of MIF inhibition on human MPM development and possibly to develop new therapies for this fatal disease.

## 4. Materials and Methods

### 4.1. Isolation of H2052/484 Cells and Cell Culture

The MPM cell lines H28 (NCI-H-28), H2052 (NCI-H2052), and MSTO (MSTO211H) were purchased from American Type Culture Collection (Manassas, Virginie, VA, USA). The MPM cell lines JL-1 was established and characterized in our laboratory from human biopsies [13]. H2052/484 cells were subcultured after mechanical dissociation of an orthotopic tumor explanted 102 days after an intrapleural implantation of 1 × 10^6^ NCI-H2052 cells into an athymic Nude-*Foxn1nu* nu/nu. All cells were routinely cultured in RPMI 1640 medium containing 10% (*v*/*v*) fetal bovine serum (complete RPMI, Life Technologies, Carlsbad, CA, USA). The cultures were grown at 37 °C in 5% CO_2_.

### 4.2. Total RNA Isolation and Real-Time RT-PCR

The expressions of *CDH1*, *CDH2*, *VIM*, *TWIST*, *SNAIL1*, *SNAIL2*, *ZEB1*, *ZEB2*, *MIF*, *CD74*, *GAPDH*, *GUSB*, *EEFLA1,* and *TBP* mRNAs were evaluated by quantitative RT-PCR analysis. Total mRNA from each cell lines was extracted by InViTrap^®^ Spin Universal RNA Mini kit (Stratec, Birkenfeld, Germany) according to the manufacturer’s instructions.

Reverse transcription and Real-time RT-PCR was performed at the same time using ONE-step kit Converter (Takyon-Eurogentec UF-RTAD-D0701, Eurogentec, Liège, Belgium) and No ROX SYBR MasterMix blue dTTP (Takyon Eurogentec UF-NSMT-B0101, Eurogentec, Liège, Belgium). Real-time RT-PCR was performed on each sample in duplicate with 50 ng cDNA per condition, using a Biorad CFX Connect Real Time system. SYBR green primer sequences for the targeted human genes are available upon request. The results were normalized to the expression levels of housekeeping genes, including *GAPDH*, *GUSB*, *EEFLA1,* and *TBP* genes.

### 4.3. Cell Growth and Vitality

Five thousand cells per well were cultured into 96-well microplates in complete RPMI. After cell adhesion, the medium was replaced, and the cells were cultured in RPMI without or with increasing concentrations of FBS for 24 h and 48 h at 37 °C. Cell growth was determined by crystal violet staining. Briefly, after fixation with formalin 10%, the cells were stained with 0.1% crystal violet (Sigma-Aldrich Corp., St. Louis, MO, USA) for 30 min at room temperature. The cells were lysed in a 10% acetic acid solution for 30 min at room temperature. The absorbance was read in a spectrophotometer at 570 nm.

Cell vitality was determined by the reduction of 3-(4,5-dimethylthiazol-2-yl)-2,5-diphenyltetrazolium bromide (MTT, Sigma-Aldrich Corp., St. Louis, MO, USA). The MTT solution (500 µg/mL in RPMI) was added for 2 h at 37 °C. The cells were lysed with dimethyl sulfoxide. The absorbance was read in a spectrophotometer at 570 nm.

### 4.4. Cell Lysis and Western Blotting Analysis

The samples were lysed in RIPA buffer (50 mM Tris, 150 mM NaCl, 0.1% SDS, 0.5% Sodium deoxycholate, 1% Igepal CA630, 2 mM EDTA, 50 mM NaF, pH 8) supplemented with a protease inhibitor cocktail (Roche Molecular Diagnostics, Pleasanton, California, CA, USA) and titrated using the DC Protein Assay (Bio-Rad Laboratories, Hercules, California, CA, USA). Amounts of 5 to 20 μg of proteins were separated by SDS-PAGE and transferred onto nitrocellulose membranes (Amersham, Little Chalfont, UK). The membranes were blocked 1 h at room temperature, incubated overnight at 4 °C with primary antibodies (EMT sampler kit, 9782 Cell signaling (Danvers, Massachusetts, MA, USA) and B-Actin, A2066 Sigma-Aldrich (Sigma-Aldrich Corp., St. Louis, MO, USA), incubated for 1 h with secondary antibodies (Bio-Rad Laboratories, Hercules, California, CA, USA), and developed using a standard ECL protocol. The quantifications were performed by using a ChemiDoc MP and the Image Lab software (Bio-Rad Laboratories, Hercules, California, CA, USA).

### 4.5. In Vivo Development of H2052/484 MPM Cells in Nude Mice

The mice were anaesthetized with isoflurane. Buprenorphin (analgesic) was injected subcutaneously. (0.05 mg/kg). MPM H2052/484 cells were injected into the left pleural cavity (1 × 10^6^ tumor cells suspended in 50 µL of RPMI) of 19-week-old athymic female nude mice nu/nu (Envigo, Huntingdon, UK) (*n* = 28). Briefly, the mouse was placed on the left side (left lateral decubitus). A 0.5 to 1 cm incision of the skin was made to expose the ribs. An amount of 50 µL of cell suspension was slowly injected into the intercostal space on the right dorsal mid-axyllary line just below the inferior border of the scapula. The wound was closed with three to four absorbable sutures. Anesthesia was stopped. The mouse was placed under a heat lamp until awake.

2-deoxy-2-[^18^F]fluoro-d-glucose ([^18^F]FDG)-PET/computed tomography (CT) scans were used to follow the intrapleural tumor growth. PET/CT was performed using a Triumph PET/SPECT/CT system (Trifoil, Chatsworth, CA, USA) on mice fasted for 12 h. An amount of 5–6 MBq of [^18^F]FDG was i.v. injected retro-orbitally on anesthetized mice. The mice were then left awake at RT during an uptake time of 60 min. Subsequently, 700 μL of 132 mg/mL meglumine ioxitalamate (Telebrix, 6% m/v iodide, Guerbet AG, Zürich, Switzerland) was injected intraperitoneally in mice to delineate the abdominal region, and the mice were subjected to CT scans. Images were obtained at 80 kVp, 160 μA, and 1024 projections were acquired during the 360° rotation, with a field of view of 71.3 mm (1.7× magnification). Sixty min after the [^18^F]FDG injection, PET scans were started for a duration of 20 min. PET scans were reconstructed with the built-in LabPET software (Triumph-Adler, Nuremberg, Germany), using an OSEM3D (20 iterations) algorithm, and the images were calibrated in Bq/mL by scanning a phantom cylinder. Reconstruction of the CT scans was performed with the Triumph XO software (Triumph-Adler, Nuremberg, Germany) that uses a matrix of 512 and a voxel size of 0.135 mm. CT scans and PET scans were co-registered using the plugin Vivid (Trifoil) for Amira (FEI, Hillsboro, OR, USA) and exported as dicom files. The software Osirix (Pixmeo, Bernex, Switzerland) was used to quantitatively analyse the datasets and generate pictures.

Two weeks after cell injection, the mice were checked every other day by observers for signs of morbidity. Euthanasia endpoints were chosen to minimize the distress of the transplanted mice. They were defined in a previous pilot study in which body weight, body condition scoring, appearance, and behavioral assessments were used to evaluate morbidity in this orthotopic mouse model of mesothelioma. The following criteria were determined: significant tumor growth and/or malignant pleural effusion in the thoracic cavity (detected by PET/CT imaging), labored breathing, abnormal posture, dehydration, and weight loss of 15% within a few days. This study was conducted under protocols revised and approved by the institutional animal care and use committee and by Geneva’s veterinarian state office.

When committee-approved endpoints (authorization GE/106/16 approved by the “Direction Générale de la Santé”, Republic of Geneva, 19 July 2016, 25291) were achieved, the mice were euthanized and closely examined for the presence of thoracic tumors. For each euthanized mouse, blood was drawn from the heart. For histology, spleen, lung, and tumors were explanted and fixed in 10% neutral buffered formalin.

### 4.6. Immunohistochemistry Analysis

The MPM samples fixed in formalin were embedded in paraffin. Four-μm-thick MPM tumor sections were cut and stained with haematoxilin-eosin (HE) or analyzed by immunohistochemistry. Labeling with anti-MIF (gift of Thierry Roger, Lausanne, Switzerland), anti-CD74 (HPA010592, Sigma-Aldrich Corp., St. Louis, MO, USA), and anti-CD44 antibodies (HPA005785, Sigma-Aldrich Corp., St. Louis, MO, USA) was performed using the Ventana Discovery automated staining system (Ventana Medical Systems, Tucson, AZ, USA). Ventana reagents for the entire procedure were used. Antigen retrieval was performed by heating the slides in CC1 cell conditioning solution for 20 min (EDTA antigen retrieval solution pH 8.4; 20 min for CD74 and CD44, 36 min for MIF). The slides were incubated 30 min at 37 °C with primary antibodies diluted at 1/300 (MIF), 1/1000 (CD74), and 1/500 (CD44) in an antibody diluent from Dako (S2022, Agilent technology, Santa Clara, CA, USA). Anti-MIF, anti-CD74, and anti-CD44 labeling was detected using the rabbit OmniMap kit (760-149). Immunostaining with anti-Ki67 (9027, Cell signaling technology, Danvers, MA, USA), γ-H2AX (sc-101696, Santa Cruz Biotechnology, Dallas, TX, USA), CD31 (ab28364, Abcam, Cambridge, UK), calretinin (18-0211, Invitrogen, Carlsbad, CA, USA), and mesothelin (HPA017172, Sigma-Aldrich Corp., St. Louis, MO, USA) was performed after EDTA antigen retrieval for 15 min. After a 20 min blocking step in PBS 0.2%/Triton X100 (PBST), the sections were incubated with primary antibodies in blocking buffer overnight at 4 °C (Ki67, 1/500; γ-H2AX, 1/500; CD31, 1/50; calretinin, 1/80; mesothelin, 1/80). The sections were washed with PBST and incubated for 1 h with DAPI 0.4 μg/mL and secondary A488-conjugated anti-rabbit antibodies in blocking buffer (A21206, Molecular Probes, ThermoFisher Scientific, Waltham, MA, USA). The slides were washed and mounted in fluorescence mounting medium (Dako, Agilent technology, Santa Clara, CA, USA). The slides were scanned with an Axioscan.Z1 and analyzed with Zen (Zen 2.3, Carl Zeiss, Oberkochen, Germany). Specific binding of all antibodies was previously checked by running controls without primary antibodies (see Supplementary Materials figure).

### 4.7. Collection of Pleural Fluid and Flow Cytometry Staining

Following euthanization, the thoracic cavity of each mice was opened and washed with 1 mL of cold sterile PBS supplemented with 3% FBS. The pleural fluid (PF) was aspirated and placed on ice before centrifugation at 300× *g* for 5 min. The supernatant was removed and stored at −80 °C. The cell pellet was washed with 10 mL of PBS/3% FBS, resuspended in 2 mL PBS/3% FBS, and carefully layered upon 2 mL of Ficoll-Paque Plus separation medium (GE Healthcare, Munich, Germany). The Ficoll gradient was centrifuged for 20 min at 400× *g* without brake. The mononuclear cells were collected, washed in PBS/3% FBS, and resuspended in 400 μL of PBS-3% FBS-1mM EDTA (FACS buffer). The cells were incubated with Fc-blocking reagent (TrueStain, Biolegend, San Diego, CA, USA) for 5 min and subsequently stained for 30 min at 4 °C with the relevant antibody. The lymphocytes were characterized with APC anti-CD19 (6D5) and FITC anti-CD45R/B220 (RA3-6B2). The antibodies used to analyze monocytes, macrophages, and MDSC were PE anti-CD11b (M1/70), PE/Cy7 anti-F4-80 (BM8), APC anti-CD206 (C068C2), and FITC anti-Gr1 (RB6-8C5). NK cells were characterized with PE anti-CD49b (DX5) (BioLegend, San Diego, CA, USA). The controls received equivalent concentrations of isotype-matched IgG. All samples were acquired with a BD Accuri C6 flow cytometer and analyzed with BD Accuri C6 and FlowJo software (FlowJo V10-CL, Tree Star Inc., Ashland, OR, USA) Monocytes/macrophages, lymphocytes, and granulocytes were first gated according to a SSC-A vs. FSC-A scatter plot, and doublets were excluded using a pulse geometry gate FSC-H × FSC-A plot.

### 4.8. Statistics

The results are presented as means ± SEM or SD as indicated. Kruskal–Wallis test was used to examine statistical differences among three or more groups. Differences between pairs of groups were examined for statistical significance using the unpaired Mann–Whitney *U* test. A *p* value < 0.05 was considered statistically significant.

## Figures and Tables

**Table 1:** Score of H2052/484 tumor development.

Score	Macroscopic Observations
1	Tumor limited to the thoracic surface of the left lung (injection site)
2	Tumor(s) on the left lung Tumor(s) 2 mm or less along - the pulmonary veins and arteries - the inferior vena cava (mediastinal pleura) No tumor on the aortic arch, the thoracic muscle, the diaphragm, and the pericardium
3	Tumors (more than 2 mm or more than 5 tumors) - on the left and right lungs or/and - along the pulmonary veins and arteries or/and - along the inferior vena cava (mediastinal pleura) Scattered foci of tumor (2 mm or less) on the thoracic muscle or/and the aortic arch No tumor on the diaphragm and the pericardium
4	Tumors (more than 2 mm or more than 5 tumors) on - the lungs or/and - the pulmonary vascular trunk or/and - the mediastinal pleura or/and - the thoracic muscles and/or the aortic arch Scattered foci of tumor (1 mm or less) on the diaphragm or the pericardium
5	Tumors (more than 2 mm or more than 5 tumors) on - the lungs or/and - the pulmonary vascular trunk or/and - the mediastinal pleura or/and - the thoracic muscles and/or the aortic arch or/and - on the diaphragm or the pericardium OR - extension of tumor from the visceral pleura into the underlying pulmonary parenchyma
6	Advanced tumors involving each of the pleural surfaces (parietal, mediastinal, diaphragmatic, and visceral pleura) with confluent pleural tumors.

**Table 2:** Immune cell number in the pleural fluids from mice with differential H2052/484 MPM development.

Tumor Development Score	0	1–3	4–6
Total pleural cells	185,942 ± 118,481	111,068 ± 35,687	425,358 ± 63,830
	*n* = 4	*n* = 10	*n* = 14
*p* vs. 0		*>0.9999*	*0.1472*
*p* vs. 1–3			***0.0021***
Lymphocyte number	36,376 ± 4135	18,779 ± 2638	96,651 ± 21,303
	*n* = 4	*n* = 10	*n* = 14
*p* vs. 0		*0.2943*	*0.3602*
*p* vs. 1–3			***<0.0001***
Granulocyte number	2085 ± 515	7025 ± 1686	33,012 ± 8879
	*n* = 4	*n* = 10	*n* = 11
*p* vs. 0		*0.6575*	***0.0037***
*p* vs. 1–3			***0.0238***
Monocyte/macrophage number	121,038 ± 93,659	81,626 ± 32,076	305,638 ± 54,706
	*n* = 4	*n* = 10	*n* = 14
*p* vs. 0		*>0.9999*	*0.1696*
*p* vs. 1–3			***0.0044***

**Table 3:** Immune cell distribution in the pleural fluids of mice with differential H2052/484 MPM development. MDSC, myeloid-derived-suppressor cells

Tumor Development Score	0	1–3	4–6
Lymphocytes (% of total cells)	41.9 ± 10.7	28.5 ± 4.8	24.6 ± 4.0
	*n* = 4	*n* = 10	*n* = 14
*p* vs. 0		*0.6644*	*0.3602*
*p* vs. 1–3			*>0.9999*
CD19^+^ B220^+^ lymphocytes (% of total cells)	8.7 ± 2.7	17.5 ± 5.3	6.8 ± 1.9
	*n* = 4	*n* = 5	*n* = 11
*p* vs. 0		*>0.9999*	*>0.9999*
*p* vs. 1–3			*0.2621*
monocytes/macrophages (% of total cells)	53.8 ± 11.2	58.7 ± 6.8	68.3 ± 4.3
	*n* = 4	*n* = 7	*n* = 14
*p* vs. 0		*>0.9999*	*0.6247*
*p* vs. 1–3			*>0.9999*
CD19^−^ CD11b^+^ monocytes/macrophages (% of total cells)	52.1 ± 10.6	53.3 ± 7.1	65.5 ± 4.7
	*n* = 4	*n* = 7	*n* = 14
*p* vs. 0		*>0.9999*	*0.9253*
*p* vs. 1–3			*0.4993*
CD19^−^ CD11b^+^ F4/80^+^ Macrophages (% of total cells)	22.9 ± 8.0	26.3 ± 10.2	45.3 ± 7.0
	*n* = 4	*n* = 7	*n* = 14
*p* vs. 0		*>0.9999*	*0.2956*
*p* vs. 1–3			*0.3193*
CD11b^+^ F4/80^+^ CD206^+^ M2 macrophages (% of total cells)	1.6		5.6
	*n* = 4		*n* = 9
*p* vs. 0			*0.4140*
Neutrophils (% of total cells)	2.2 ± 0.6	8.9 ± 1.7	7.8 ± 1.5
	*n* = 4	*n* = 10	*n* = 14
*p* vs. 0		***0.0224***	***0.0456***
*p* vs. 1–3			*>0.9999*
Neutrophil-to-lymphocyte ratio	0.06 ± 0.01	0.38 ± 0.06	0.36 ± 0.06
	*n* = 4	*n* = 10	*n* = 11
*p* vs. 0		***0.0095***	***0.0206***
*p* vs. 1–3			*>0.9999*
CD49b^+^ NK cells (% of total cells)	5.5 ± 1.9	7.6 ± 1.3	7.0 ± 1.7
	*n* = 4	*n* = 7	*n* = 12
*p* vs. 0		*>0.9999*	*>0.9999*
*p* vs. 1–3			*>0.9999*
CD11b^+^ Gr1^+^ MDSC (% of total cells)	27.7 ± 6.7		51.1 ± 8.1
	*n* = 4		*n* = 9
*p* vs. 0			*0.1063*

## Supplementary Materials

Supplementary materials can be found at http://www.mdpi.com/1422-0067/19/7/1881/s1.

## Abbreviations

MPM	Malignant pleural mesothelioma
MIF	Macrophage migration inhibitory factor
MTT	3-(4,5-dimethylthiazol-2-yl)-2,5-diphenyltetrazolium bromide
FBS	Fetal bovine serum
FDG	Fluoro D glucose
PET/CT	Positron emission tomography/computed tomography
HE	Haematoxylin-eosin
NK	Natural killer
MDSC	Myeloid-derived-suppressor cell
FSC	Forward scatter
SC	Side scatter
