# Supplementary material for: Experimental Model of Human Malignant Mesothelioma in Athymic Mice
Source: Int J Mol Sci. 2018 Jun 26;19(7):1881. doi: 10.3390/ijms19071881 (PMC6073357; doi:10.3390/ijms19071881)
Supplement: Supplementary file 1 [file ijms-19-01881-s001.pdf]

Supplementary figure

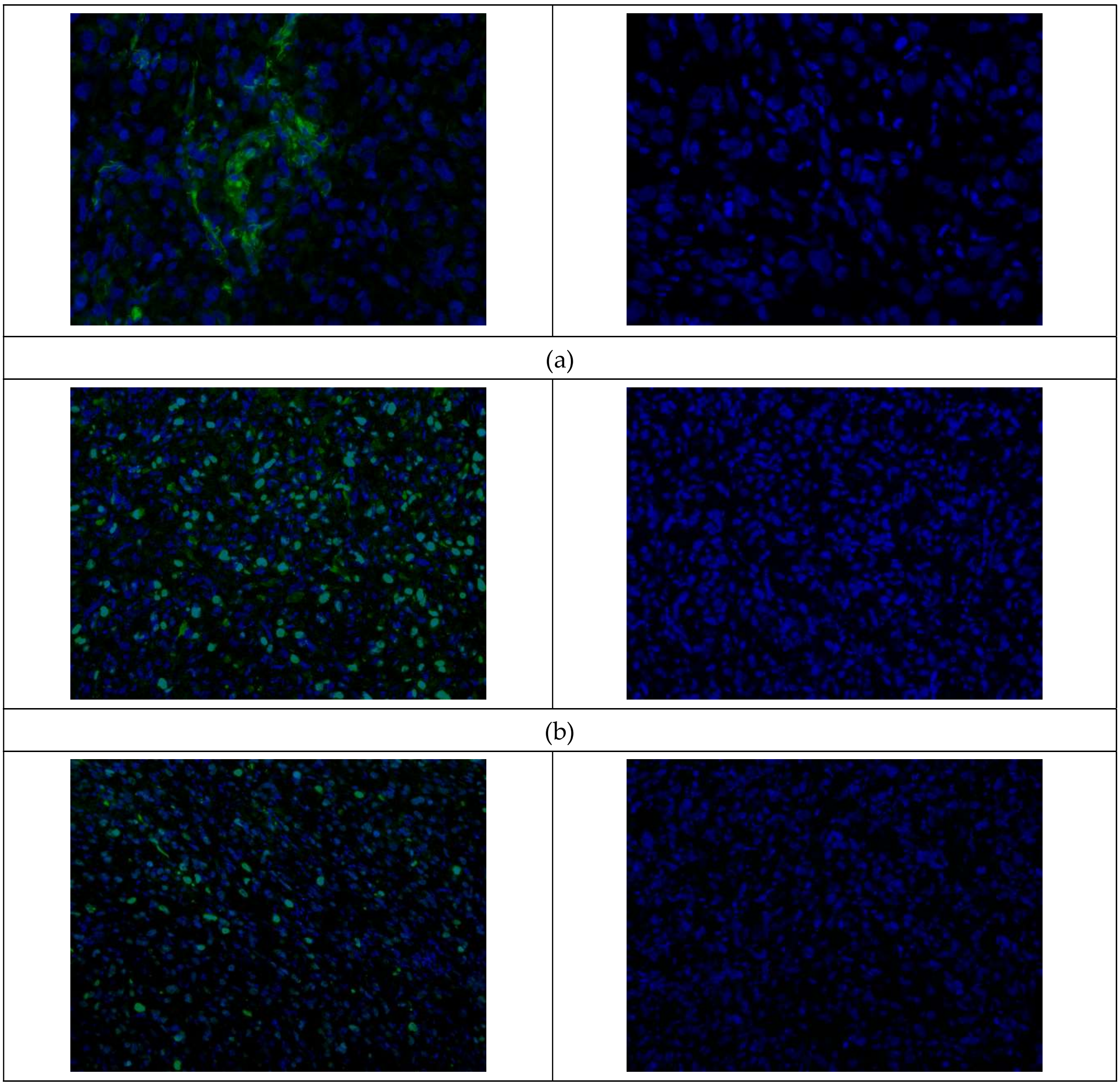

(c)

Supplementary figure. Staining of consecutive H2052/484 tumor sections performed with 1st and 2nd antibodies (left; positive control) or without the 1st antibody (right; negative control) **(a)** anti-CD31 (vascularization), **(b)** anti-Ki67 (cell proliferation), and **(c)** anti-γ-H2AX (DNA damage and cell apoptosis).
